# The consequences of *Brugia malayi* infection on the flight and energy resources of *Aedes aegypti* mosquitoes

**DOI:** 10.1038/s41598-019-54819-2

**Published:** 2019-12-05

**Authors:** Alastair G. T. Somerville, Katherine Gleave, Christopher M. Jones, Lisa J. Reimer

**Affiliations:** Liverpool School of Tropical Medicine, Pembroke Place, Liverpool, L3 5QA UK

**Keywords:** Animal behaviour, Animal physiology, Entomology

## Abstract

Evidence from experimental infection studies has shown that infected mosquitoes exhibit altered host-seeking behaviours, with suppression and activation of behaviours dependent on the parasite’s development stage. The mechanisms are poorly characterised; however, infections can impact mosquito energy reserves, thereby influencing key life-history traits and behaviours. In addition, filarial infection is likely detrimental to flight due to damage caused by developing worms. This study aimed to evaluate the impacts of *Brugia malayi* infection on *Aedes aegypti* flight parameters: distance, average speed, maximum speed and number of flight bursts, using a tethered flight mill. In addition, we explored whether differences in flight capacity may be due to the effect of infection on glycogen and lipid reserves. Infection with filarial worms significantly reduced flight distance but increased the number of flight bursts. Exposure to microfilaermic blood led to a significant decrease in average and maximum flight speeds even in the absence of an established infection. Mosquitoes fed on microfilaraemic blood showed reduced levels of glycogen (−37.9%) and lipids (−49.7%) compared to controls at nine days post-exposure. However, a one-hour period of flight activity caused an increase in lipid content for both infected and control mosquitoes. Consequential flight incapacitation may serve in explaining the heterogeneous distribution of lymphatic filariasis.

## Introduction

Lymphatic filariasis (LF) is a parasitic disease caused by three nematode species: *Wuchereria bancrofti*, *Brugia malayi* and *B. timori*^1,2^. Regarded as a Neglected Tropical Disease (NTD), recent estimates suggest that 67 million people suffer from LF^3^ across 73 tropical and sub-tropical countries^4^. Morbidity, largely in the form of elephantiasis and hydrocele, causes substantial physical, social and psychological damage^5–7^. The resulting loss of 2.8 million disability-adjusted life years (DALYs)^8^ places LF as a significant global health problem. Microfilariae (mf) released by gravid female worms are ingested by mosquitoes during bloodfeeding. Approximately 10 days later, following successive developmental stages in the flight muscles, the infective larvae (L3) migrate to the mouthparts of the mosquito, ready to be deposited onto vertebrate host skin in subsequent feeds. This mode of development causes substantial histological and physiological damage within the vector^9–13^. As such, filarial infection is likely to be detrimental to mosquito flight.

Evidence of the changes in mosquito flight following filarial infection are equivocal. Previous work suggests that flight activity, when defined as the number of minutes within an hour containing at least a single flight attempt, increases three-fold during filarial infection prior to the development of L3 worms^14^. Conversely, some studies suggest that filarial infection produces significant declines in continuous measures of flight ability, namely distance and time^15–18^. Comparing study outcomes is therefore difficult due to differences in measured flight variables; an issue which is further complicated considering that there are a range of tools with which to quantify flight. Therefore, while infection may indeed lead to an increased number of flight attempts but overall reduced flight outputs, no study to date has explored both aspects of flight ability and activity.

Lipids and glycogen are important sources of energy in mosquitoes, being utilised in a number of life history traits and behaviours, such as flight^19,20^, vitellogenesis^20^ and immune responses^21,22^. The relative availability of these resources may therefore influence flight ability or activity. Blood-fed and sugar-fed mosquitoes have been shown to differ in flight speed and distance^20^, perhaps due to the differential utilisation and availability of energy resources. Research which has shown reduced flight distance and time as a result of filarial infection^18^ hypothesised this was a result of depleted energy reserves, however no study to date has explored energy in infected mosquitoes.

Mosquitoes infected with *Plasmodium* show alterations in host-seeking and host-feeding strategies depending on the stage of infection^23–28^. Similar changes in host-seeking behaviour have been observed in mosquito-filarial systems, including *Aedes aegypti* mosquitoes infected with *B. malayi*^29^. When harbouring the non-infective L2 stage, mosquitoes show a five-fold reduction in host-seeking following exposure to human host cues compared to uninfected controls. Once developed into L3s, this pattern is reversed, and infected mosquitoes are significantly more likely to respond to host cues than uninfected mosquitoes^29^. These behavioural changes offer clear advantages to the parasite in minimising mortality risk during development and increasing the chances of host-contact once transmissible. The cause of this altered host seeking behaviour remains unclear, however damage to the flight muscles caused by the developing filarial worm may inhibit or deter flight in mosquitoes prior to the L3 stage.

Behavioural and physiological changes in vectors following infection can have significant implications for disease transmission^30,31^, and modelling frameworks are likely to benefit from an increased understanding of the interaction between vector and parasite, particularly during infection^32^. However, the absolute fitness costs associated with infection in mosquitoes remain ambiguous^33^. Coevolution between obligate parasites and vectors may lead to neutral relationships to maintain effective transmission, but immune responses are costly, and parasites can cause direct physical damage to the insect^9–13^.

The primary aim of our study was to determine the influence of filarial infection on a range of mosquito flight parameters: distance (m), speed (ms^-1^), maximum speed (ms^-1^) and number of flight bursts (justifications for which can be seen in Table 1). Our secondary aim was to determine whether infection also led to a depletion of glycogen and lipid resources, and if this depletion was associated with flight performance.

**Table 1:** The definition and rationale for the flight responses measured and analysed using the tethered flight mill system.

Flight Parameter	Unit	Definition	Rationale
Flight Distance	Meters (m)	The total distance covered over one hour.	Damage caused to thoracic flight muscles by developing filarial worms is likely to affect flight distance. Previous studies indicate reduced distance from filarial infection^18^.
Average Speed	Meters per second (ms^−1^)	The average (harmonic mean) distance covered per second across one hour.	Damage caused to thoracic flight muscles by developing filarial worms is likely to affect measures of flight speed.
Maximum Speed	Meters per second (ms^−1^)	The highest speed reached within flight testing	Damage caused to thoracic flight muscles by developing filarial worms is likely to affect measures of flight speed.
Number of Flight Bursts	—	Any flight attempt that lasts more than 5 seconds and covered a distance of at least 0.25 m^*^	Previous studies indicate reduced flight attempts following filarial infection^14^.

^*^Definition of a flight burst was based on pre-existing definitions of a flight burst^65^.

## Results

A total of 217 female *Ae. aegypti* mosquitoes (including 123 fed *Brugia malayi* and 94 fed uninfected blood) were flown on tethered flight mills across three replicate experiments. Midgut dissections conducted 3 hours post-bloodfeeding confirmed substantial uptake of microfilariae (mf) in mosquitoes which fed on *B. malayi* infected blood (n = 3, $\bar{{\rm{x}}}$ = 109.7 ± 10.7). Post-flight dissections of mosquitoes which fed on *B*. *malayi* infected blood found that 41/65 (63.1%) were infected with L1s and/or L2s at 4 to 6 days post-exposure (DPE), and 29/58 (50.0%) were infected with L3s at 11 to 13 DPE (Table 2). We categorised mosquitoes into three groups based on infection status: “Infected” (fed on infected blood and contained at least one worm at the time of dissection), “Exposed” (fed on infected blood but contained no worms at the time of dissection), and “Control” (fed on uninfected blood).

**Table 2:** Numbers of mosquitoes assayed for flight and the intensity of *B. malayi* infection.

Days Post Exposure	Infection Status^†^	Sample Size	Total Number of Worms Recovered	Mean Intensity (95% CI)
**4–6**	Infected	41	185	5.08 (3.79–6.37)
	Exposed	24	0	0
	Control	48	—	—
**11–13**	Infected	29	108	4.10 (3.53–4.68)
	Exposed	29	0	0
	Control	46	—	—
Total	**217**	**293**	—	

^**†**^Exposed mosquitoes were fed the same bloodmeal as infected but were found not to contain larvae after flight testing.

### Effect of *B. malayi* infection on mosquito flight

**Figure 1:**
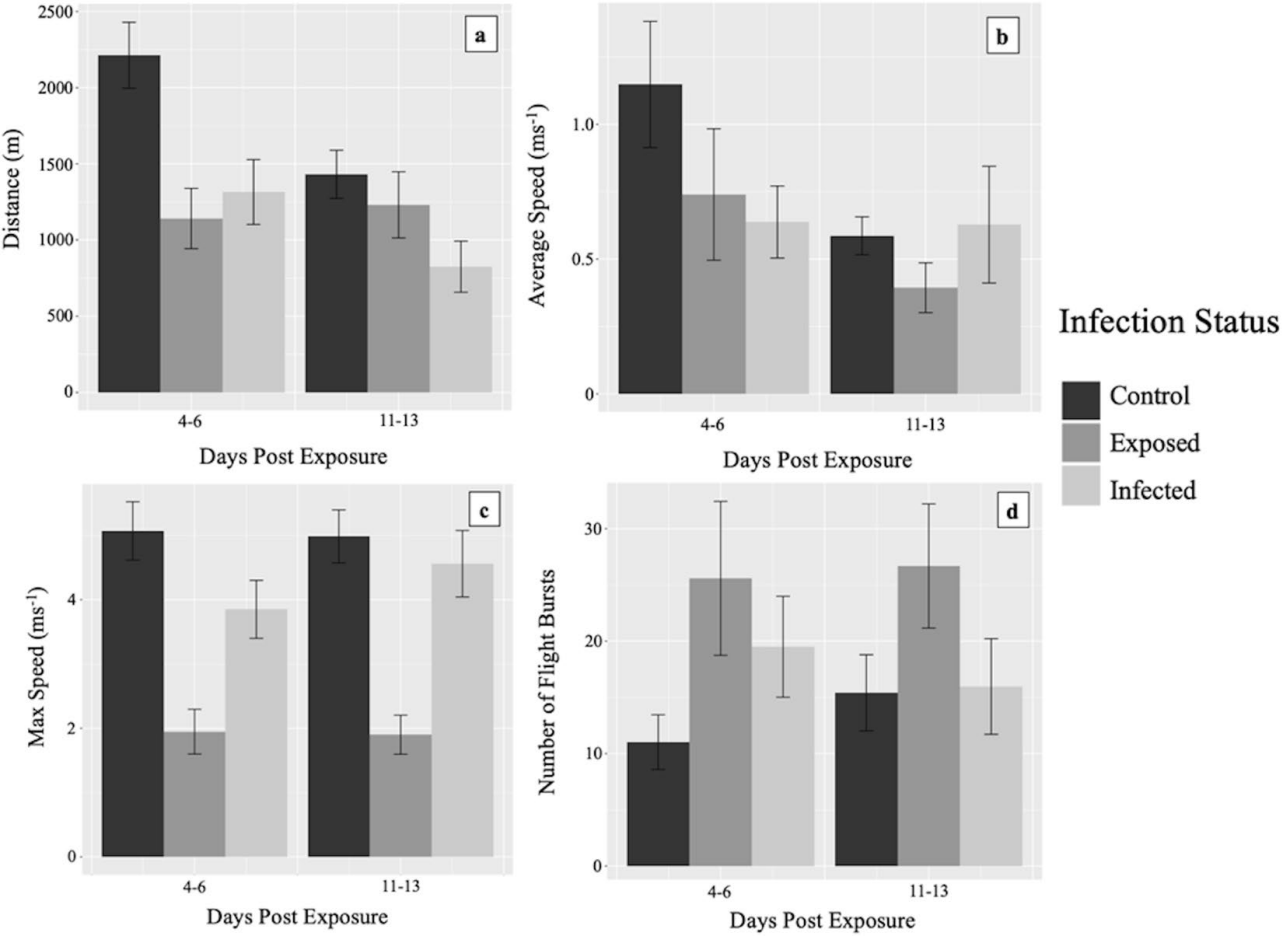
The relationship between *B. malayi* infection status and flight activity in *Ae. aegypti* mosquitoes post-exposure. (**a**) Distance (m), (**b**) average speed (ms^-1^), (**c**) maximum speed (ms^-1^), (**d**) number of flight bursts. All mosquitoes were flown for a total time of one hour. Standard error bars are shown. Exposed mosquitoes were fed the same bloodmeal as infected but were found not to contain larvae after flight testing.

Generalised Linear Mixed Models (GLMMs) using likelihood ratio testing indicated that infection status had a significant effect on flight distance (χ^2^ = 10.5, *P* = 0.005), average speed (χ^2^ = 10.3, *P* = 0.006), maximum speed (χ^2^ = 20.5, *P* < 0.001) and the number of flight bursts (χ^2^ = 17.6, *P* < 0.001). Pairwise comparisons based on least square means found that infection and exposure both lead to declines in the distance and average speed flown, as well as an increase in the number of flight bursts when compared to controls (see Table 3). The number of days post-exposure only had a significant impact on flight distance and the number of flight bursts, with flight distance decreasing significantly by 11–13 DPE (χ^2^ = 6.4, *P* = 0.012), whereas the number of flight bursts increased significantly (χ^2^ = 16.2, *P* < 0.001). A significant interaction between infection status and DPE was observed for the number of flight bursts (χ^2^ = 120.3, *P* = < 0.001). This indicates that the change in the number of flight bursts over time between infection groups was different. The unadjusted means for each measured parameter are shown in Fig. 1. Wing length measured from a total of 34 mosquitoes found the average wing length to be 3.101 ± 0.022 mm. Scatter-plots of wing length against measured parameters of flight found no correlation (Supplementary Fig. S1). Linear regression analysis also found that worm burden was not a significant predictor for any flight parameter, with the exception of maximum speed during the L3 stage (*P* = 0.001), which increased with increasing worm burden (Supplementary files S2 and S3).

**Table 3:** Results of Generalised Linear Mixed Models on the effect of *B. malayi* infection status on mosquito flight.

Flight response	Independent variable	Pairwise comparison†	Estimate	Std. Error	p-value
Flight distance (m)	Infection status	Control - Exposed	0.109	0.180	0.817
		Control - Infected	0.497	0.151	0.003**
		Exposed - Infected	0.388	0.188	0.097
	DPE	—	−0.337	0.132	0.012*
Average speed (ms^−1^)	Infection status	Control - Exposed	0.544	0.165	0.003**
		Control - Infected	0.210	0.145	0.318
		Exposed - Infected	−0.334	0.170	0.120
	DPE	—	−0.236	0.128	0.067
Max speed (ms^−1^)	Infection status	Control - Exposed	0.618	0.134	<0.001***
		Control - Infected	0.230	0.109	0.087
		Exposed - Infected	−0.389	0.144	0.019*
	DPE	—	0.018	0.092	0.849
Number of flight bursts	Infection status	Control - Exposed	0.002	0.045	0.999
		Control - Infected	0.162	0.045	<0.001***
		Exposed - Infected	0.159	0.043	<0.001***
	DPE	—	0.138	0.034	<0.001***

DPE = Days Post Exposure.

*P < 0.05; **P < 0.01; ***P < 0.001.

^†^Exposed mosquitoes were fed the same bloodmeal as infected but were found not to contain larvae after flight testing.

### Effect of *B. malayi* infection on lipid and glycogen content in mosquitoes

A total of 76 mosquitoes underwent glycogen and lipid content analysis using anthrone and vanillin reagent, respectively. Midgut dissections conducted 3 hours post-bloodfeeding confirmed uptake of mf in *B. malayi* exposed female mosquitoes (n = 6, $\bar{{\rm{x}}}$ = 32.3 ± 9.12). Feeding on *B. malayi* positive blood was found to have a significantly detrimental effect on glycogen (χ^2^ = 6.0, *P* = 0.014), decreasing it by 37.6%, and lipid (χ^2^ = 28.2, *P* < 0.001), decreasing it by 49.7%. Due to the nature of content extraction, infection could not be confirmed in these mosquitoes.

### Effect of flight on lipid and glycogen content in mosquitoes

**Figure 2:**
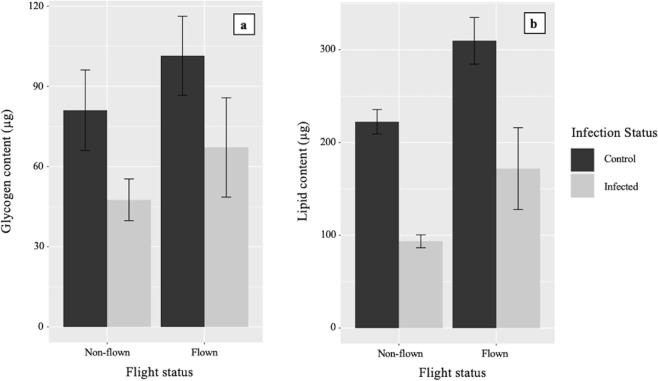
The glycogen and lipid content of *Ae. aegypti* mosquitoes based on *B. malayi* mf feeding status and flight status. (**a**) Glycogen, (**b**) Lipid. All mosquitoes were allowed to fly for a total time of one hour. Mosquitoes are categorised as either controls, or having fed on infected blood, as confirmation of infection intensity was not possible. Standard error bars are shown.

Flight activity had no significant effect on glycogen levels (χ^2^ = 2.3, *P* = 0.132), but did lead to significantly increased lipid content (χ^2^ = 13.3, *P* < 0.001) of 34.7% (Table 4). Changes in the average glycogen and lipid levels between groups are shown in Fig. 2.

**Table 4:** Effect of *B. malayi* infection and flight on energy resources.

Energy resource	Independent variable	Estimate	Std. Error	p-value
Glycogen	Infection status	−0.468	0.205	0.014*
	Flight status	0.287	0.205	0.132
Lipid	Infection status	−0.717	0.162	<0.001***
	Flight status	0.473	0.162	<0.001***

*P < 0.05; ***P < 0.001.

## Discussion

This study found that *B. malayi* infection has a detrimental impact on *Ae. aegypti* flight distance, average speed and maximum speed. Previous research has hypothesised that decreased flight capabilities were the result of depleted host energy reserves and/or functional incapacitation of flight muscles^18^. *Ae. aegypti* muscle fibres become devoid of glycogen granules when infected with developing filarial larvae due to consumption by the worm^10,11^. Immune responses in insects are also energetically costly^34,35^, and infection can lead to significant declines in glycogen and lipid content in both *Drosophila*^36,37^ and *Ae. aegypti*^38^. Upregulation of lipid transporter proteins during *Ae. aegypti* infection suggests an increased utilisation of lipids during systemic immune responses^21^. The declines in flight outputs observed in this study may therefore be a consequence of energy resource deprivation resulting from costly immune responses, and/or the direct consumption of intramuscular glycogen by the filarial worm. This is supported by the significant declines of glycogen and lipid content in *Brugia*-infected mosquitoes observed here.

Insect flight is one of the most energetically demanding exercises in the animal kingdom and requires highly efficient systems to transport energy reserves to flight muscles^39^. Glycogen and lipids are the primary sources of energy for insect flight, including mosquitoes^40,41^, but their levels did not decrease following the one-hour flight period in this study. In the wild, mosquitoes derive their energy from the nectar of a range of available plant sources before it is converted to glycogen and storage lipids over a period of two days^20^. The cohorts of *Ae. aegypti* were provided with 10% glucose solution up until flight testing and therefore declines in glycogen levels following flight may not have been observed due to the preferential use of glucose. Conversely, why lipid content increased following flight is not immediately clear. Short-term stress, such as flight, can lead to liberation of lipids from the fat body^42–44^, a phenomenon shown in a number of different insect species^45–48^. Lipids are stored as triacylgycerols in the fat body, before being converted to diacylglycerol during liberation and transportation in the haemolymph^49^. There is evidence that vanillin reagent, the choice of reagent for this study, fails to react with triacylglycerols^50^, perhaps suggesting that lipids were only detectable once liberated from the fat body for flight.

Interestingly, infection was associated with an increase in the number of flight bursts, suggesting that infected vectors may make a larger number of slow, short, flight attempts. This finding lends its support to previous research which has found *Plasmodium* infection is associated with an incapacitation of flight^51,52^, but increased nectar-feeding^53^. Reduced energetic reserves caused by harbouring an infection support this idea, although further research is needed. Conversely, it may simply be that the presence of parasitic worms agitates the invertebrate host, leading to increases in the number of flight bursts.

Physical damage from filarial worm development appeared to have little to no additive impact on the flight ability of mosquitoes. Mosquitoes undergo age-associated declines in flight activity^54^, which was also observed in our study. However, the proportional decrease in distance and average speed over time was less in mosquitoes infected with L3 larvae compared to controls. The detrimental effect of infection on flight ability may therefore occur rapidly following exposure. Previous studies which identified significant declines in flight ability and activity following the development of L3s did so using unnaturally high burdens of infection (10+ worms)^14^. Thus, while high burdens of infection may influence flight at the infective stage, this has limited applicability to natural settings, where burdens of infection rarely exceed five L3 worms per host^55,56^. Flight muscle fibres can carry out post-trauma reparation if such damage is limited^10^, further supporting this idea that L3s only cause noticeable detriment to host flight in unnaturally high intensity infections. Previously identified stage-specific switches in host-seeking behaviour^29^ are unlikely to be due to the generic effects of flight capacity, however it could be partially attributed to filarial infection causing an apparent increase in the number of flight bursts. Control mosquitoes in this study otherwise saw a relative decline in the number of flight bursts over time.

The distinction between infection and exposure (the absence of developing or infective larvae) on flight outputs is not obvious. While exposure appeared to match infection in its effect on flight distance and average speed, it had a significantly greater detriment to maximum speed than infection. Regardless of the reason for this difference, these results clearly highlight that exposure and clearance of filarial worms may be sufficient to cause significant changes in vector flight.

## Conclusion

This study highlights the impacts that filarial infection can bear on vector flight. Further investigations into the flight behaviour of infected mosquitoes is necessary to apply this to different ecological settings, however the interplay between infection, immunity and flight is clearly complex. Furthermore, understanding the impact of fitness costs on the ability of mosquitoes to transmit disease may help explain the heterogeneity of filariasis transmission^57^. The behavioural and physiological consequences of filarial infection on their invertebrate hosts, such as flight incapacitation, may contribute to the heterogeneous nature of LF, which can pose a challenge for elimination. Remarkable differences between mosquito species in their behavioural and physiological responses to infection warrants continued exploration with additional parasite-vector systems.

## Methods

### Study design

We tested the impact of filarial infection on various flight parameters in mosquitoes using a set of eight tethered flight mills. *Ae. aegypti* Liverpool (LVP) strain mosquitoes which had fed on either microfilaermic blood or uninfected blood were flown at 4 to 6 days post-exposure (DPE) or 11 to 13 DPE, corresponding to the L1/L2 and L3 stages of *B. malayi* respectively^58^. Those which fed on blood containing *B. malayi* mf underwent subsequent dissections to confirm the prevalence and intensity of infection. The wing length of 34 infected mosquitoes was measured to determine the correlation with flight activity. Mosquitoes were subject to either (i) a one-hour flight mill assay on day 4–6 DPE followed by dissection, (ii) a one-hour flight mill assay on day 11–13 DPE followed by dissection, (iii) a one-hour flight mill assay on day 9 DPE followed by glycogen and lipid analysis, or (iv) glycogen and lipid analysis on day 9 DPE with no flight. A single energy content analysis was conducted to observe the impact of infection and flight on glycogen and lipid reserves.

### Mosquito rearing and husbandry

Rearing of the *Ae. aegypti* LVP mosquitoes took place at the Liverpool School of Tropical Medicine (LSTM) insectaries under controlled conditions (80% relative humidity, 27 °C and 12:12 light/dark cycle). Internally sourced mosquito egg papers were floated out into plastic trays (235 × 345 × 75 mm) containing distilled water, with a larval density of approximately 200/larvae per tray. Larvae were fed on Brewers’ yeast pellets before transfer to cages (285 × 295 × 280 mm) once pupated. We maintained adults on 10% sugar solution prior to bloodfeeding. All adult females were between 2 and 5 days old when bloodfed.

### Mosquito exposure to blood sources

On the day of bloodfeeding, female mosquitoes were split into two separate cages. We then fed cohorts on either uninfected human blood (controls) or human blood containing *B. malayi* mf at a density of 20,000 mf/ml. Mf were obtained via intraperitoneal lavage of infected gerbils by a third party, in accordance with UK Home Office requirements and following LSTM approval, before suspension in RPMI media at a dilution of 1/100. Triplicate microscopic observations confirmed the presence and mobility of mf. Uninfected control blood was diluted with equal measures of non-infected RPMI media. Approximately 3 ml of blood was then offered to mosquitoes using a Hemotek® membrane feeding system kept at 37 °C. Following successful bloodfeeding, we removed all mosquitoes that had not fed to repletion. All research red cells and plasma were supplied by National Health Service Blood and Transfusion and were mixed in a 50:50 ratio before use. To verify mf uptake, three engorged females had their midgut contents homogenised on a microscope slide, which was then scanned with phase optics at 10x magnification.

### Quantification of flight ability

**Figure 3:**
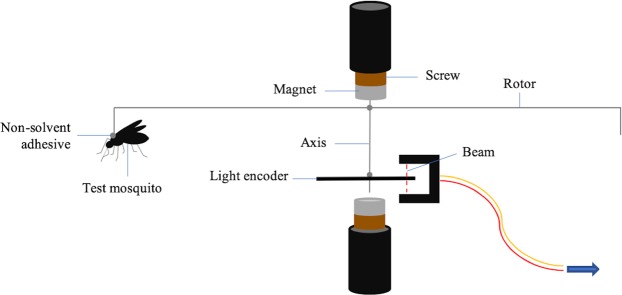
The set-up of a flight mill used during testing in this study, including rotor. Mosquitoes fly around a radius measuring 4 cm, causing the light encoder to periodically break a laser beam which measures distance. 1 rotation = 25.13 cm. Image provided by A. Somerville.

We assessed the flight activity of *Ae. aegypti* using tethered flight mills (provided by Dr. Lim of Rothamsted Research), which were housed under standardised insectary conditions described above (Fig. 3). Females were briefly knocked down on ice and placed on a metal plate with their dorsal surface showing. The arm of a flight mill rotor (radius = 4 cm) was dipped in non-solvent fast-drying glue, before being placed onto the scutum and held for approximately one minute to allow the glue to dry. Mosquito orientation was kept horizontal and perpendicular to the rotor bar. Successfully adhered mosquitoes were allowed to rest with tarsal contact prior to placement into one of eight flight mill chambers, which holds the central steel axis of the rotor in place by two opposing magnets to minimise friction. Individuals were briefly observed to ensure initiation of flight before being left to fly freely for one hour. Mosquitoes which failed to fly when stimulated were removed and replaced. The distance covered every 5 seconds to the nearest 10 cm is automatically recorded on software (Flight Mill v1.2) according to flight mill design. All flight mill experiments were performed in LSTM insectaries under rearing conditions and occurred between 0900 and 1700. We chose to measure a total of four flight activity response variables to determine the impact of infection on flight performance. The definitions and justifications for these are provided in Table 1.

### Dissections of mosquitoes to categorise infection

Dissections of mosquitoes corresponded to the developmental time points of *B. malayi*. At 4 to 6 DPE, mosquito thoraces were separated from the body in *Aedes* saline^59^ on a microscope slide, and the number of worms present was counted on a stage microscope as described for mf uptake verification. At 11 to 13 DPE, the abdomen, thoraces and heads were all separated from individual mosquitoes and placed in *Aedes* saline. Each body part was then broken into large defined pieces and left for approximately two minutes. The number of L3 was counted under a dissection microscope. We considered mosquitoes which had fed on microfilaraemic blood but contained no worms as “Exposed” during analysis.

### Energy content analysis

Nine days post-feeding, glycogen and lipid content were analysed. Glycogen and lipids were extracted using the standardised methods as described by Van Handel^60,61^ and quantified using anthrone and vanillin reagent respectively. A 96 well plate held triplicate aliquots of 0.2 ml extract from each mosquito sample, and photospectrometry optical density (OD) readings at 625 nm quantified contents. Triplicate OD readings were carried out using a plate spectrophotometer and recorded on software (Gen5 Version 2.04) before averaging. Standard curves of lipid and glycogen content were created using olive oil suspended in chloroform and anhydrous glucose suspended in de-ionized water, respectively. Both standard solutions included 100 mg of substrate dissolved in 100 ml of liquid. Using the standard curve equations, OD readings of glycogen and lipid content from the mosquito samples were converted to quantity readings (μg) prior to statistical analysis.

### Statistical analysis

All statistical analysis was conducted in RStudio (version 1.1.1456)^62^ using the lme4 (version 1.1–20) package^63^ and visualised using ggplot2 (version 2.2.1)^64^. Statistical analysis of flight ability was conducted using GLMMs. Prior to analysis, Wald test assessments of the random variables (the flight mill number and replicate) found one of the flight mills to be faulty, so individuals flown on this flight mill (n = 24) were omitted from further analysis. Individuals which flew <50 m were also not included as it was assumed that attachment to the flight mill had compromised their flight. Linear regression analysis found that wing length did not correlate with any flight parameters, and so it was removed as a random variable in models. We assessed the distribution of each flight variable by plotting a histogram of individual mosquito flights and modelled them accordingly using GLMMs. Likelihood ratio testing (LRT) between individual GLMMs tested for an effect of either infection status or DPE on each flight variable. A least square means approach for multiple comparisons with a Tukey adjustment (α = 0.05) tested for differences between infection status categories. All best fit models were assessed using residual-fitted plots and/or Normal Q-Q plots to ensure reliability. LRT between Generalised Linear Models (GLMs) tested for associations between energy reserves and flight and infection status.

## Supplementary information

Supplementary Figures
