# Supplementary material for: The consequences of Brugia malayi infection on the flight and energy resources of Aedes aegypti mosquitoes
Source: Sci Rep. 2019 Dec 5;9:18449. doi: 10.1038/s41598-019-54819-2 (PMC6895159; doi:10.1038/s41598-019-54819-2)
Supplement: Supplementary file 1 — Supplementary Figures [file 41598_2019_54819_MOESM1_ESM.pdf]

The consequences of *Brugia malayi* infection on the flight and energy resources of *Aedes aegypti* mosquitoes

Alastair G.T. Somerville, Katherine Gleave, Christopher M. Jones, Lisa J. Reimer

Supplementary Files

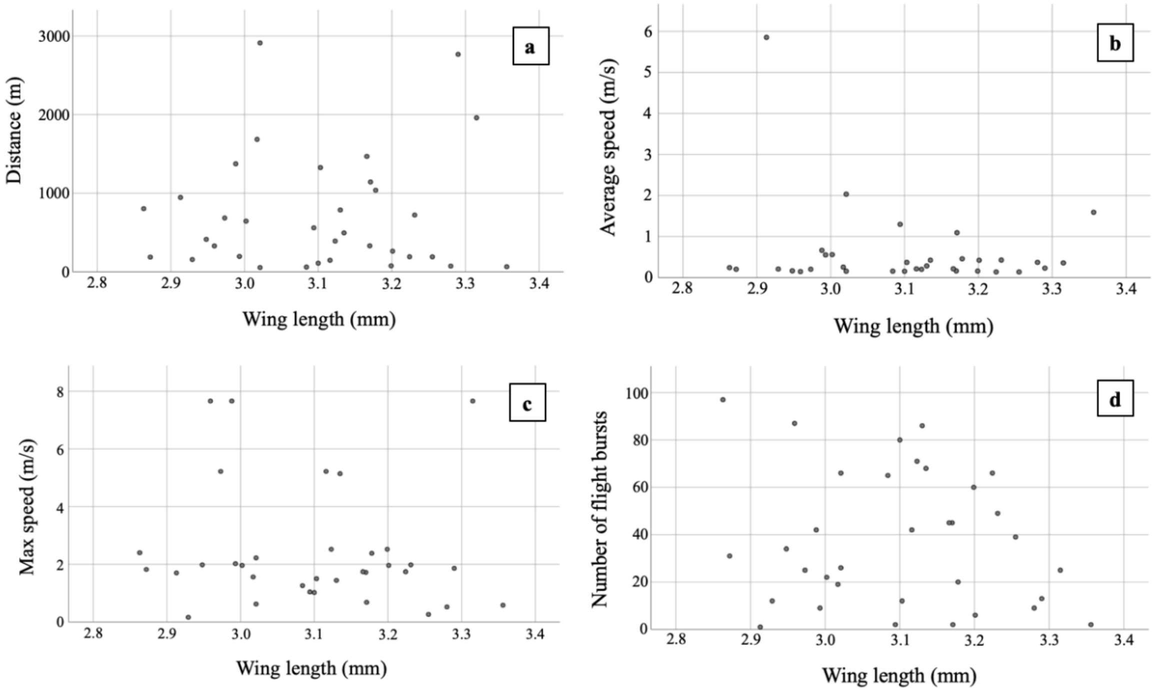

Supplementary figure 1. Scatter-plots of flight parameters against wing length. a) Distance, b) Average speed, c) Max speed, d) Number of flight bursts.

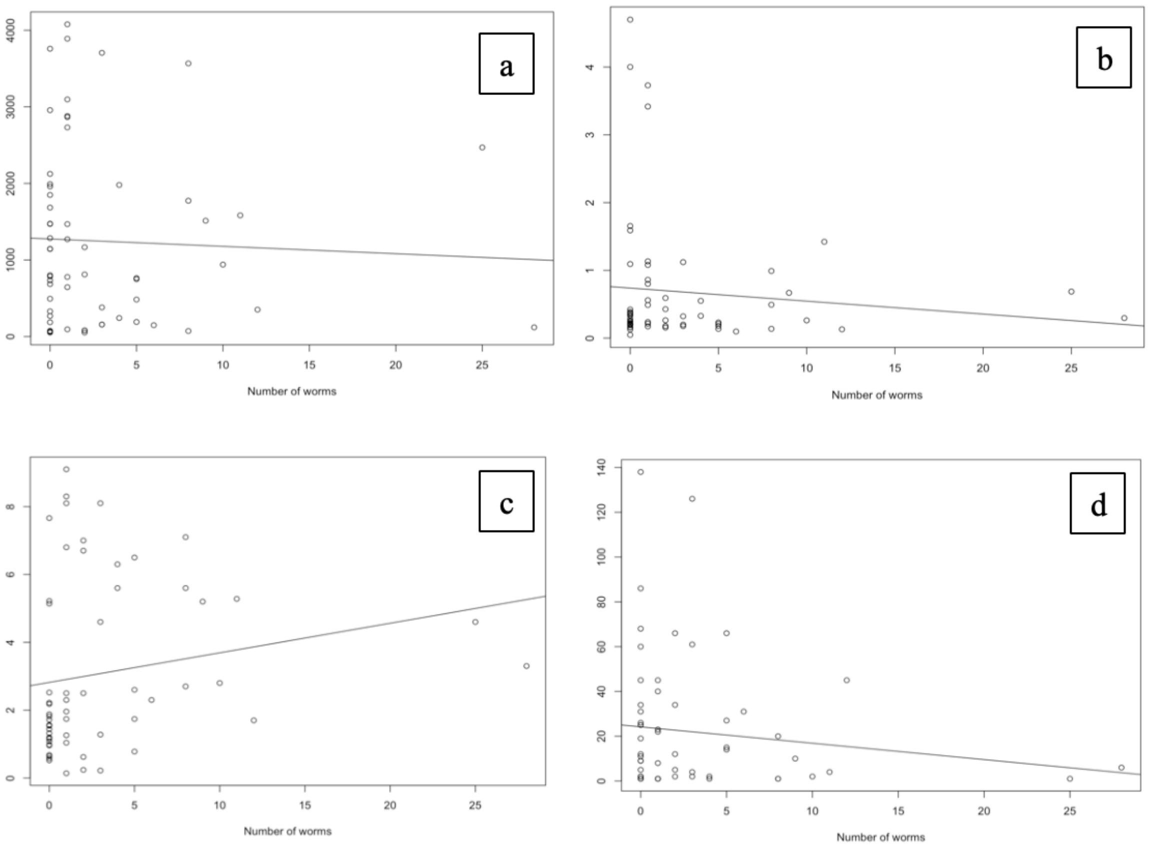

Supplementary figure 2. Linear regression plots of measured flight parameters against worm burden from 4 to 6 DPE. a) Distance, b) Average speed, c) Maximum speed, d) Number of flight bursts. For no variable was the linear regression result significant.

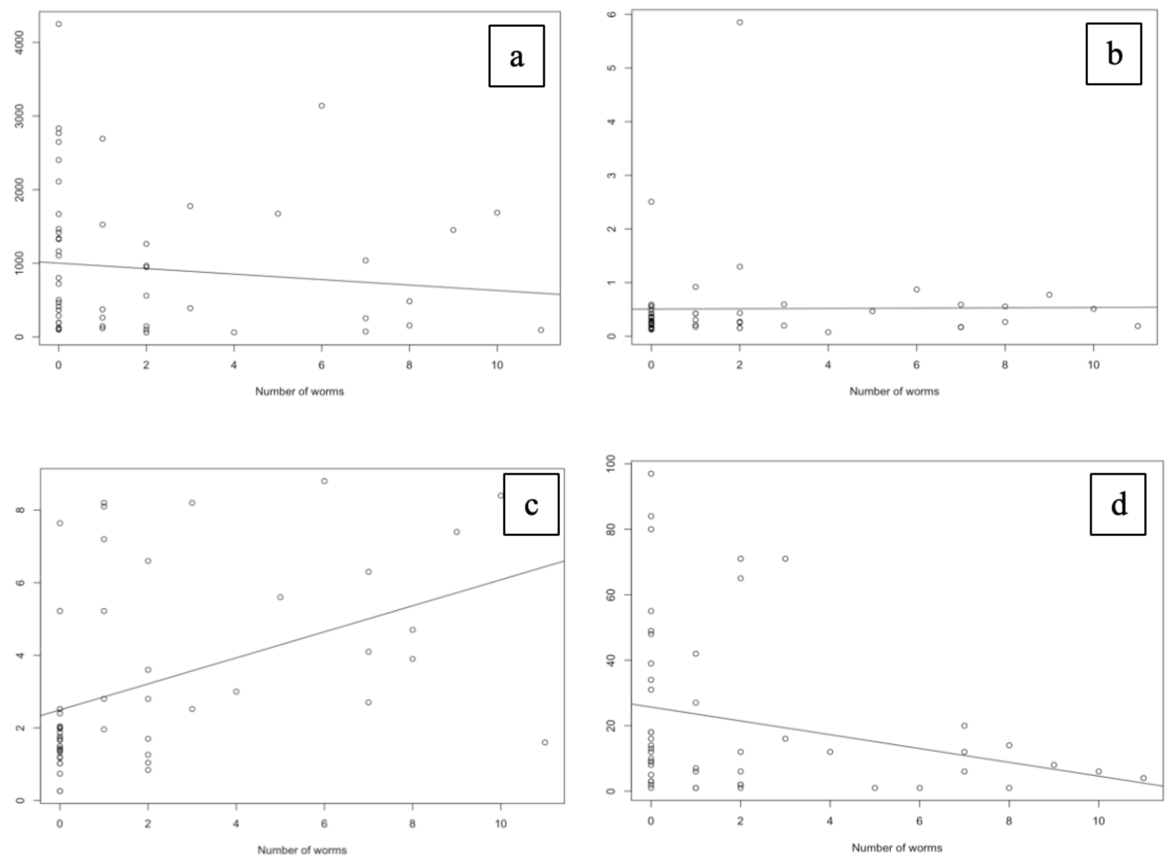

Supplementary figure 3. Linear regression plots of measured flight parameters against worm burden from 11 to 13 DPE. a) Distance, b) Average speed, c) Maximum speed, d) Number of flight bursts. Worm burden was found to be a significant predictor of maximum speed only (*P*=0.001).
